# Amaurosis and Retro-Bulbar Neuritis

**Published:** 1904-10-01

**Authors:** 


### Hospital Clinics.

### AMAUROSIS AND RETRO-BULBAR NEURITIS.

Loss of sight without appreciable change in the eyeballs was in pre ophthalmoscopic days a comparatively common occurrence, and such cases, without much attempt at explanation or analysis, were grouped under the term *amaurosis.* So long as the retina, the optic nerve, the choroid, and the deeper media were beyond the reach of direct observation, it was not possible to appreciate in these structures changes which produced blindness, and therefore, so long as the superficial and visible parts of the eyeball retained their normal appearance, loss of sight was a fact that hardly admitted of exact interpretation. The entire extent of the visual path from the retina to the cerebral centres was open to accidents that could not be discovered, and there was no guide to explain the nature and site of the lesion. Hence the name amaurosis had frequently to be invoked as a convenient cloak for an extensive range of ignorance. Thanks to more modern methods of observation, it is now known that many cases of blindness which formerly were named but not understood depend on definite changes in the fundus oculi, and to a corresponding extent, therefore, the use of the term amaurosis has been reduced. It has given place to such exact diagnoses as retinitis, retinal detachment, optic neuritis, choroiditis, embolism, and the like. Nevertheless, there still remain certain cases of loss of sight for which the ophthalmoscope discovers no explanation. Probably the best known of these are the instances which occur in the course of kidney disease, where at times and without obvious cause the patient becomes completely blind. It is to be presumed that here the condition is a functional one due to the action of poisons retained in the blood on the visual centres. The fact that, together with the blindness, such symptoms as headache, vomiting, convulsions, etc., are usually present supports this view, as does also the restoration of sight as the uræmic symptoms disappear. There is yet another piece of evidence which points in the same direction—namely, the frequent retention in these cases of the pupillary light response. Obviously this fact shows that the condition or lesion responsible for the blindness must affect some part of the visual path posterior to the corpora quadrigemina, and this alone brings the visual centres under suspicion. Had the hypothetical poison which caused the uræmic phenomena affected through the blood supply the peripheral distribution of the optic nerves—as is theoretically possible—it is manifest that the pupil light reaction must have disappeared. This is the more interesting when placed in contact with the fact that large doses of quinine have been known to cause blindness; but here, unlike what occurs in uræmic poisoning, the action of the poison would appear to be spent on the peripheral part of the visual apparatus. For in such instances examination with the ophthalmoscope shows considerable spasm of the retinal arteries, as well as pallor of the optic disc, and thus it may be presumed that the functions of the retina are suspended as a consequence of deficient blood supply due to arterial spasm. Sodium salicylate is another drug which has been known to cause blindness, presumably by much the same method as that involved in quinine poisoning. And in both cases the restoration of sight is comparatively prompt (granted the discontinuance of the drug), an experience which harmonises with the view that the effect on the nerve structures is not direct but indirect—that is, through changes produced in the blood supply. As is well known, tobacco is also an agent which produces failure of sight with little or no manifest retinal changes. Here it is believed a definite nerve poison is at work, and in some cases an appreciable pallor of the temporal half of the disc may be recognised. It is presumed that tobacco has a specially poisonous influence on the papillomacular bundle of nerve fibres, and hence it is found that central vision more particularly suffers and the patient has a blind area (scotoma) at the centre of the visual field. Exactly the same condition is sometimes observed in cases of diabetes, and here pathological examination has demonstrated a particular strand of degenerated nerve-fibres through the course of the optic nerve. Another poison capable of producing similar results is methyl alcohol, and indeed it is more than possible that ordinary ethyl alcohol has its share in producing cases diagnosed as tobacco amblyopia. Many examples of blindness due to methyl alcohol have now been recorded, several of them being of an acute character. Examples of blindness due to drinking essence of Jamaica ginger have been reported from time to time, but it is fairly certain that the toxic agent in the "essence" was not the ginger but methyl alcohol. Now in all the above instances of blindness without obvious ophthalmoscopic changes the affection involves the sight of both eyes, because the defect in vision arises from the action of a poison circulating in the blood and having equal access to the structures on each side of the middle line. A poison thus distributed must inevitably have bilateral effects whether its action falls on the cerebral centres or on peripheral structures. Hence failure of sight in both eyes, with normal appearances in each fundus oculi, suggests at once the presence of a poison in the blood. It is far different when the impairment of vision is restricted to one eye. Here the manifest presumption is in favour of a local lesion, and in view of the anatomical association of the nerve fibres from the two eyes in the optic commissure and in the optic paths posterior to the commissure, it is manifest that failure of vision in one eye only must mean a lesion in the visual path anterior to the commissure. In such a case, if there are no changes in the structures of the eyeball, there is no site for the lesion other than the trunk of the optic nerve. The ophthalmoscope shows that the lesion is not in the retina or optic disc; the restriction of the visual defect to one eye proves it cannot be in or behind the optic commissure. It must, therefore, lie between these two extremes; that is, in the portion of the optic nerve (retro-bulbar) behind the eyeball. Now cases of one-eyed blindness, with such results on examination as have just been defined, do occasionally occur, and are usually grouped as examples of retro-bulbar neuritis. In such cases there may often be observed facts other than the above, which suggest that the pathological lesion is situated in the trunk of the optic nerve. Thus the pupil light response on the affected side is either lost or is seriously defective; often, whilst there is a sharp contraction at the outset, this is only momentary, and almost at once gives place to full dilatation. Again, there is frequently pain on lateral movement of the eyeball, as if this strained some part in a condition of undue irritation. And, once more, it is not infrequent in such cases to observe that whilst at the outset the optic disc has a perfectly normal appearance, later on the whole or part of the disc becomes unduly white, just as if a lesion in the nerve behind the globe had led to a degenerative or sclerotic change which, travelling along the nerve fibres, had ultimately reached the optic disc itself. Thus, whilst blindness in both eyes developing without appreciable ophthalmoscopic change means the presence of some poison in the blood, a one-eyed blindness with similar attendants means a lesion in the post-bulbar portion of the optic nerve on the affected side. The nature of such a lesion, it must be admitted, is somewhat obscure. The suddenness with which the blindness in these cases usually develops suggests at once that the lesion is vascular in origin, and the pain on movement of the eyeball already referred to may be quoted in proof of its inflammatory nature. Certain it is that the phrase retro-bulbar neuritis is advanced as the appropriate diagnostic title. Even if the accuracy of this be allowed, however, an explanation of such an occurrence is very difficult. In some of these cases there has been exposure to a cold wind shortly before the onset of the blindness, and thus it has been suggested that the condition is parallel to a Bell's paralysis occurring in like circumstances. In other instances, however, no such history has existed. Syphilis is another explanation which has been advanced, but it certainly does not apply to the majority of the cases. One of the most interesting associations of retro-bulbar neuritis is disseminated sclerosis. It is quite beyond dispute that in a certain proportion of cases a retro-bulbar neuritis has been followed by the development of this disease. The interval between the two may be a long one, but the cases in which the sequence has been observed are too numerous to be dismissed as mere coincidences. Granting the view that a retro-bulbar neuritis depends on a vascular lesion, its occasional association with disseminated sclerosis has a further interest, when it is remembered that the modern view of this disease is that it depends on patches of inflammation in the cerebro-spinal axis produced by an infective agent present in the blood. What determines the locality of these patches is quite uncertain, but it is notorious that their distribution often departs widely from a pattern of bilateral symmetry. Hence the almost invariably unilateral development of retro-bulbar neuritis does not necessarily remove it from a clinical and pathological relationship to disseminated sclerosis. And its usually sudden development is obviously in harmony with the inflammatory and vascular theory of that disease. But to all this it is necessary to add the remark that not every case of retro-bulbar neuritis develops at a later stage into disseminated sclerosis. It is a clinical event which, more especially in the case of a young adult, brings the nervous system under suspicion, but beyond this it is not safe to advance. In a fair proportion of the cases the eyesight is regained in perfect measure; in others there is more or less permanent damage; whilst in a third group the eye remains practically blind. But, whatever be the immediate result, it is certain that many patients who have suffered an attack of retro-bulbar neuritis continue to enjoy good health for many years after the event. Their nervous systems do not show any evidences of disseminated sclerosis or of other form of organic disease. Thus the prognosis, both immediate and remote, in any case of retro-bulbar neuritis is one of much difficulty. If recovery from the loss of sight is to take place, signs in this direction can usually be appreciated within a fortnight or less, and so long as these are absent the prospect is uncertain. With the individual patient it would seem unnecessary to mention the possibility of disseminated sclerosis in the entire absence of all other suggestions of that disease. Rest for the eyes, potassium iodide or iodide of iron, and perhaps blisters to the temple, comprise the main measures of treatment and often gain a striking success. Yet it must be allowed that retro-bulbar neuritis remains somewhat of a mystery, and, though amaurosis as a cloak for ignorance has, thanks to the ophthalmoscope, a much diminished opportunity, there yet remain cases of blindness which present a riddle hard to be read.

